# How Can Hybrid Simulation Support Organizations in Assessing COVID-19 Containment Measures?

**DOI:** 10.3390/healthcare9111412

**Published:** 2021-10-21

**Authors:** Chiara Cimini, Giuditta Pezzotta, Alexandra Lagorio, Fabiana Pirola, Sergio Cavalieri

**Affiliations:** Department of Management, Information and Production Engineering, University of Bergamo, 24044 Dalmine, Italy; giuditta.pezzotta@unibg.it (G.P.); alexandra.lagorio@unibg.it (A.L.); fabiana.pirola@unibg.it (F.P.); sergio.cavalieri@unibg.it (S.C.)

**Keywords:** hybrid simulation, COVID-19, agent-based simulations, discrete event simulation models

## Abstract

Simulation models have always been an aid in epidemiology for understanding the spread of epidemics and evaluating their containment policies. This paper illustrates how hybrid simulation can support companies in assessing COVID-19 containment measures in indoor environments. In particular, a Hybrid Simulation (HS) is presented. The HS model consists of an Agent-Based Simulation (ABS) to simulate the virus contagion model and a Discrete Event Simulation (DES) model to simulate the interactions between flows of people in an indoor environment. Compared with previous works in the field of simulation and COVID-19, this study provides the possibility to model the specific behaviors of individuals moving in time and space and the proposed HS model could be adapted to several epidemiological conditions (just setting different parameters in the agent-based model) and different kinds of facilities. The HS approach has been developed and then successfully tested with a real case study related to a university campus in northern Italy. The case study highlights the potentials of hybrid simulation in assessing the effectiveness of the containment measures adopted during the period under examination in the pandemic context. From a managerial perspective, this study, exploiting the complementarity of the ABM and DES approaches in a HS model, provides a complete and usable tool to support decision-makers in evaluating different contagion containment measures.

## 1. Introduction

Coronaviruses (CoVs) are a large family of respiratory viruses such as MERS (Middle East Respiratory Syndrome) and SARS (Severe Acute Respiratory Syndrome). They are common in several animal species but can evolve to infect humans and spread between people in rare cases. At the end of 2019, a novel coronavirus designated as SARS-CoV-2 (or COVID 19) emerged in the city of Wuhan, China, and caused an outbreak of unusual viral pneumonia [1].

Millions of people have been infected in a short period, hundred of thousands have died, and the economic losses have reached totals that are gigantic, even for macroeconomists used to big numbers [2].

Many European countries have responded to the COVID-19 pandemic by implementing nationwide protection measures and lockdowns in spring 2020. However, the epidemic rebounded in autumn 2020 and spring 2021 when such measures were relaxed, possibly leading to second or more repeated lockdowns [3].

In parallel, different private and public organizations have to take enormous strategic and organizational decisions within a highly complex system of interdependent virological, behavioral, legal, social, and economic factors.

COVID-19 affects all organizations, and it is evident that fighting this disease goes far beyond the healthcare arena. It’s high complexity and dynamics call for reliable decision-bases, not only at a national level, but also at a single organization level. Each wrong decision, even inside a single organization, can have large-scale epidemiologic and economic consequences.

From this perspective, it is essential to understand how decision-makers in each organization can re-design their internal processes to avoid the wrong decisions.

If we analyze the scientific efforts carried out up to the time this paper was written, we notice that most authors have focused their attention on the epidemiological modeling of the spread of COVID-19, via computer simulation, within nations or in a complex system. At the moment, no research has focused on how modeling and simulation techniques can be used to understand the spread of the disease inside limited closed spaces such as single organizations and help companies make better decisions for the organizational processes re-design. The modeling environment provides the unique opportunity to test and analyze different scenarios in a laboratory setting rather than experimenting on the real population. Moreover, it helps understand both the COVID-19 spread and the containment measures efficacy at an organizational and ecosystem level.

For these reasons, this study aims to answer the following research question: “How can simulation support decision-makers in re-organizing people flows and internal processes evaluating the risk of COVID-19 infection?”.

In order to answer such a question, we present the results of the adoption of Hybrid Simulation (HS) modeling as an effective approach to support organizations in assessing people flow re-design decisions to contain the spread of COVID-19. In particular, by combining the potentials of Discrete-Event Simulation (DES) to reproduce the behavior of people moving in a specific environment and the Agent-Based Simulation (ABS) to imitate the contacts and contagion between people, this paper develops a novel approach to model and evaluate individuals’ behaviors in time and space domains, modeling at the same time different phases of the COVID-19 infection. 

The HS model aims at providing a valuable tool to support decision-makers in evaluating the risks of COVID-19 infection in relation to different adopted contagion containment measures. To highlight the potential of hybrid modeling, an application case has been developed with the software Anylogic 8.7.1 to represent the spread of COVID-19 inside the buildings of an Engineering University campus in the north of Italy. The case study also has the function to check and validate whether the containment measures adopted effectively prevent contagion in the university environment.

## 2. Background

### 2.1. Epidemiological Studies

The disease is an integral part of human history. Since humans began to organize themselves in societies, contagious diseases have played a central role, strongly conditioning the birth and development of modern medicine and laying the foundation of epidemiology [4]. Epidemic modeling aims at understanding the spread of diseases in host populations, both in time and space. The first epidemiological models appeared at the beginning of the twentieth century, but many epidemiological models studying different infective diseases have been developed since the mid-1950s. Nowadays, many studies use mathematical modeling in the epidemiological field in the scientific literature, explicitly representing the epidemic dynamics [5]. These studies help evaluate different infection control interventions, assess the burden of infection, and further understand their epidemiology. Among them, fourteen studies published since 2004 have been selected and analyzed to conduct a short literature overview for identifying the most used epidemiological approaches (Table 1).

Generally, all the selected studies simulate a homogeneous reference population; however, each of them sets different assumptions about the key epidemiological parameters, such as the base reproduction numbers (R0), the incubation period, the latency period, and the infectious period, according to the epidemic treated and the developed scenario. For what concerns the studies about SARS and SARS-CoV-2 (Table 2), several common characteristics are valuable and replicable in the hybrid model developed in this paper. First, some of the studies assume that a significant amount of outbreak transmissions are asymptomatic or pre-symptomatic, introducing a new parameter: the asymptomatic transmission ratio. The asymptomatic transmission ratio refers to the percentage of infections that occurred before symptoms appeared. In detail, three studies use a range of estimates of the asymptomatic transmission ratio from 0 to 11% in their transmission models.

Moreover, in these three studies, the delay of quarantine (i.e., the time lapse between the identification of infected individuals and the implementation of the quarantine for contacts) and the delay of isolation (i.e., the time interval between the onset of symptoms and diagnosis, hence the formal hospital isolation of infected individuals) are used. Moreover, an efficiency factor is incorporated into the models. The efficiency factor represents the overall quarantine and isolation efficiency in transmission control [20].

### 2.2. Simulation Advantages for COVID-19 Containment

Simulation techniques have been successfully applied to treat several aspects of healthcare (e.g., [21,22,23]).

Recent studies applied Process Simulation to model different aspects of the COVID-19 pandemic as well. Simulation is revealed to be an effective method to assess different kinds of decisions with different levels of abstractions. A simulation is an ideal tool for assessing the re-design of the people flows within an organization to contain the COVID-19 spread and efficiently and effectively manage the internal processes to keep the organization working. In particular, the main simulation advantages are listed below:

- Simulation allows the investigation of many different process configurations in one model, regardless of how complex they are. This aspect allows us to model even very complex organizations in which different parallel processes coexist, and several different people flows intersect.
- Simulation supports “what-if” analyses. “What-if” analysis can provide insights into an existing or a hypothetical situation, allowing for safe, replicable, and usually less expensive performance tests [24]. This aspect can allow analyzing in advance the processes considering the evolution of the pandemic and the government decisions, trying to understand how to re-plan organizations as external variables change.
- Simulation can allow the analysis and measurement of the dynamic performance of a given process and the assessment of both the presence and relevance of any grouping or area at risk of contagion.
- Simulation offers interactive and visual assistance for modeling the process and the flow of people, facilitating the comprehension of the model and of its results, enhancing the validation process [25].

As underlined by authors [26], four simulation modeling methods have been adopted to different model aspects of the pandemic: System Dynamics (SD), Discrete Event Simulation (DES), Agent-Based Modelling (ABM), and Hybrid simulation (HS). The selection of the proper decision-making tools should be based on the process characteristics, situations and problems, and decisions that must be addressed.

SD dates back to 1950 and is primarily used to analyze the dynamics of a system [27,28]. In SD, the real-world processes are represented as stocks and flows between these stocks. It supports the highest level of abstraction [29]. SD modeling has demonstrated considerable value to support decision-makers in comprehending and forecasting complex systems’ behavior in support of effective policy actions. In healthcare, it has been used to study policy-level topics such as population flow, obesity, workforce demand, and HIV/AIDS [30,31].

DES is arguably the most used technique in practice [32]. It is process-centric and focuses more on the tactical/operational dimension, based on entity/people flows, resource sharing, and sequences of activities: entities can only have a passive behavior. It supports a medium–low abstraction [29,33,34]. Many applications have been made in healthcare over the last 40 years. The literature contains many DES models for patient flow, clinics, emergency departments, operating theatres, intensive care units, and hospital bed capacity management [35]. In addition, DES has been also widely used to model human movement since this is central to representing the flow of people/customers/pedestrians in a system [36].

ABM is a recent approach, more effective in modeling individuals’ behavior through a bottom-up perspective in which agents have their own rules and become active elements of the model [37]. In ABM, the global system behavior is not defined; it emerges from many individuals, each following their own rules [29]. ABM can be used to simulate people living in a population sharing a predefined environment, each one with specific attributes and behaviors.

Next to the three ‘pure’ methodologies, HS grew out of the need to combine the features and the advantages of two or more of these approaches, integrating the specific features from the different techniques [38]. This characteristic allows attaining higher flexibility and working at different levels of abstraction, exploiting each method’s strengths [39].

### 2.3. Simulation Approaches for Re-design Organization People Flow during the COVID-19 Pandemic

The single organization must re-design the internal process to reduce the risk of COVID-19 contamination, be compliant with the national and local regulations defined to reduce the COVID-19 spread, and, at the same time, be efficient and effective in the delivery of the organization core activities. Hereafter, the different ways simulation approaches can support organizations in better assessing their processes based on analyzing the main critical features characterizing the COVID-19 pandemic are discussed, considering each approach’s characteristics. In particular, the different simulation approaches are used to reach the following different aims:

- *Model a variety of possible individual infection phases and individual interactions.* ABM responds directly to the necessity of defining individual rules and behavior and describes a decentralized system as agents that can behave independently from one another. ABM has the advanced capability of tracking the movement of disease and the contacts between each individual in a population located in a predefined area. Agents would ideally work in representing various human beings in different infection phases. ABM can be used to study and track the movement of infected individuals and their contacts in a social system. In a DES, where entities (individual) can only have a passive behavior, the interaction between them can be defined a priori and can be represented by interacting with the individual. Based on stocks and continuous flows rather than on discrete entities, SD can describe interactions only at a high level of abstraction.
- *Describe inner behavior uncertainties for the individual.* The use of stochastic variables in DES allows simulating process-related uncertainties within the process of an organization (e.g., customer arrival rate, duration of activities, reliability of resources) [33], while ABM allows extending the uncertainty to human behaviors. Based on differential equations, SD may not be the best choice [29].
- *Model individual actions and organizational processes in time and space.* Both DES and ABM can respond to these. In DES, individual modeling is mainly related to their discrete action over time, but it can also capture spatial factors [40], while ABM can be used to model individuals as active agents if the modeler wants to give a higher relevance to their active interaction within the system [37]. The high detail level of SD would not fit the scope. The spread of human epidemics strongly relies on individual social networks and relations within the population. Therefore, modeling the environment where the discrete individuals interact makes it possible to understand spatial characteristics in spreading dynamics.
- *Monitor and evaluate the spread of the contagion within different control policies*. The ABM approach can be designed to allow epidemiological researchers to do a preliminary “what-if” analysis to assess systems’ behavior under various conditions and to evaluate which alternative control policies to adopt in a predefined time and space to fight epidemics. DES can be used to model and evaluate different control policies applied in organizational processes or individual actions.

The analysis of these requirements suggests that ABM could be a well-performing approach to manage several epidemiological diffusion criticalities. However, organizations require an understanding of the diffusion of the epidemic within its processes in time and space to foster at the same time COVID-19 containment and efficiency and responsiveness of the internal processes. This aspect would push the modeler towards DES due to its process-oriented nature that can better fit the modeling of processes and activities. On the contrary, an SD model could support strategic decision-making, giving insights into long-term dynamics, but the analysis shows that it might not be the best choice for the analysis at an operational level. 

ABM and DES appear complementary; therefore, a possible solution is adopting hybrid simulation in which agents can be used to model the individual’s health status and infections and their interactions, while the organization processes and individual actions can be described through DES.

Concerning the utilization of simulation techniques to model and simulate different scenarios of the COVID-19 pandemic, in the literature, several works have been published since the beginning of 2020. Table 3 presents some of these simulation studies, namely the most suitable for comparison with our study. For each of them, the simulation paradigm used (ABM, SD, DES) and the study’s objective are described and which of the above mentioned aim is actually met by the study.

As shown in Table 3, evaluating the related works that use simulation to approach the COVID-19 in consideration of the four aims mentioned above about simulation for re-designing people flows, it appears that each work has been focused only on some of the most relevant aspects of the virus spread modeling. In particular, SD works are strong in analyzing the general impacts on the population contagion and the economic effects of the pandemic, but they lack the microscopic perspective of individuals entirely. On the other side, ABM simulations can effectively reproduce the behaviors of single individuals that pass through different stages of disease development, but they are not able to represent precise movements within spaces. The DES works correctly to model this latter aspect but, concerning the current applications to the COVID-19 pandemic that have been found in the literature, they have not been used to support the re-organization of internal company processes concerning different contagion containment measures, lacking the possibility to reproduce different infection stages. Only one HS work has been reviewed, showing interesting results about the fruitful combination of the DES and ABM approaches, even if in the presented research, limitations about the modeling of processes in both time and space still exist.

In consideration of all these related works, combining the strength of ABM and DES, this study aims to cover all the four essential aspects needed to support the re-organization of people flows and internal processes in the COVID-19 pandemic (i.e., Model a variety of possible individual infection phases/interactions, Describe inner behavior uncertainties for the individual, Model individual actions and organizational processes in time and space, Monitor and evaluate the spread of the contagion within different control policies).

## 3. Methodology

**Figure 1:**
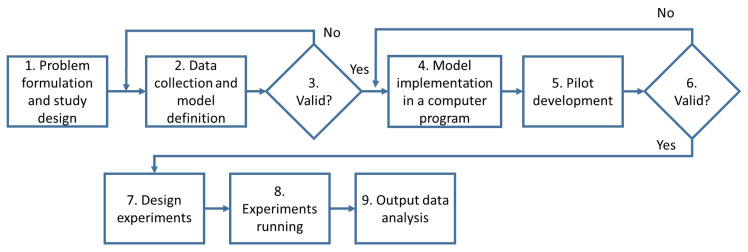
Simulation steps (authors’ elaboration from [48]).

The guidelines described by [48] have been taken as a reference to conduct the simulation case study. The iterative steps of the simulation study are represented in Figure 1. The work proposed in this paper uses Anylogic 8.7.1 software to deploy the simulation model. More information about the software and the run specification will be provided in Section 4.2.

According to the guidelines provided by [38] showed in Figure 1, the following steps have been followed:

1. *Problem formulation and study design*: the problem under investigation is the assessment of people flows management to contain the spread of COVID-19 in closed environments such as work and study places. In order to face this problem, it was decided to apply a Hybrid Simulation (HS) model. The reasons behind this choice are extensively explained in Section 2.3. The HS integrates an ABM to figure the spread of the virus and a DES to model the interactions of flows of people moving in closed environments interacting with each other (see Section 4 for details).
2. *Data collection and model definition*: in this step, we started to collect the data necessary to define the ABM and the DES models better to include them in the HS model. In particular, in order to define the details of the agent-based model, it was necessary to include all the variables involved in the spread of the virus, which are already present both in the scientific literature and in the recommendations of national and international bodies, such as the infection index, the percentage of symptomatic individuals, the incubation period and the contagion probability (reported in the Section 4.1). Then, to define the discrete event simulation model that models the interactions between flows of people, it is necessary to have people’s paths within the environments, the plans of the buildings included in the study, and the infection rules (reported in the Section 4.2). Once all the variables and parameters included in the models were defined, a first validation was needed.
3. *Validation*: validation should be done throughout the entire simulation study [38], in particular, in the building of the model it is necessary to interact with decision-makers to check the reliability of the parameters’ values set in the model. We check our choices by interacting both with an epidemiologist (for checking the epidemiology values) and with a university campus manager (for checking the policies and retrieving data about course composition and the availability of the spaces).
4. *Model implementation in a computer program*: the models were then implemented in the software used as a tool for integrated simulation, in our case Anylogic 8.7.1.
5. *Pilot development*: as a pilot model, a university campus case study was chosen for which all the data were known (see Section 5.1).
6. *Validation*: the pilot model had been tested with a sensitivity analysis fully described in Section 5.2.
7. *Design experiments*: once the pilot model had been tested, different experiment settings were designed to understand how the model changes when certain elements that strongly influence the spread of the virus were changed.
8. *Experiment running*: the model’s behavior was analyzed when the percentage of classrooms occupied and the efficiency of the protection provided by the masks varied (see Section 5.3 for more details).
9. *Output data analysis*: the results were analyzed, providing valuable indications both for the containment of Covid-19 on the University campus analyzed (and for future research directions as reported in Section 6).

More details concerning every step of the methodology are provided in the following section of the paper.

## 4. The Hybrid Simulation Model

The new Coronavirus responsible for COVID-19 respiratory disease can be transmitted from one individual to another through close contact with a probable or confirmed case. Individuals that are part of an organization are involved in a sequence of daily activities. Some of these activities occur within the organization locations (e.g., schools, workplaces, commercial shops) and may require interactions between individuals. The analysis of the activities performed by individuals belonging to the organization is essential to understand their path of movement through space and time.

The COVID-19 propagation modeled in this study considers individuals’ interactions within the organization space and performing activities during the organization’s scheduled time. To simulate the physical environment and interactions between individuals, two sub-models have been developed:

- The first sub-model was designed to describe the generic COVID-19 infection model: in this perspective, a SEIR agent-based model has been developed and adopted;
- The second sub-model was conceived to represent the flow and actions of the individual within the organization, leading to a description of the space of individual interactions while performing their activities. For this purpose, a discrete-event simulation has been adopted.

### 4.1. COVID-19 Infection Model

When a sick person coughs, sneezes, talks, and sings, the contaminated secretions (such as saliva) are released from the mouth or nose. People in close contact (less than 1 m for more than 15 min) with an infected person can become infected if the droplets enter his mouth, nose, or eyes [49].

**Figure 2:**
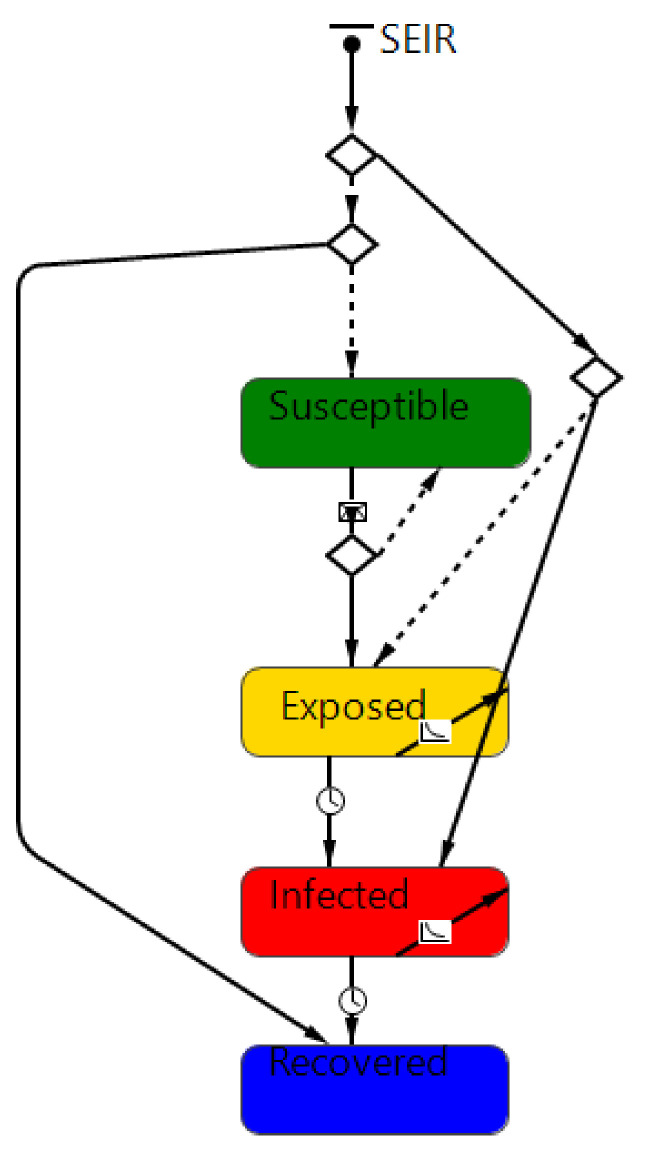
The general SEIR model.

As suggested by many authors [50,51], this simulation adopted the Susceptible-Exposed-Infectious-Removed (SEIR) model (Figure 2). In such a model, the population is divided into compartments, which represent different states concerning the disease progression of an individual: susceptible (S), exposed (E), infectious (I), and recovered (R). Susceptible individuals may become exposed if they interact with an exposed or infectious individual (β is the rate of becoming exposed if you stand with an exposed or infected person). From Exposed, the individual progresses to being infectious (I) and eventually recovered (R). An initial α probability of having exposed people and a γ probability of having infected people entering the system. There is also a rate δ of people recovered in the population, who cannot be infected by anyone (Table 4).

**Figure 3:**
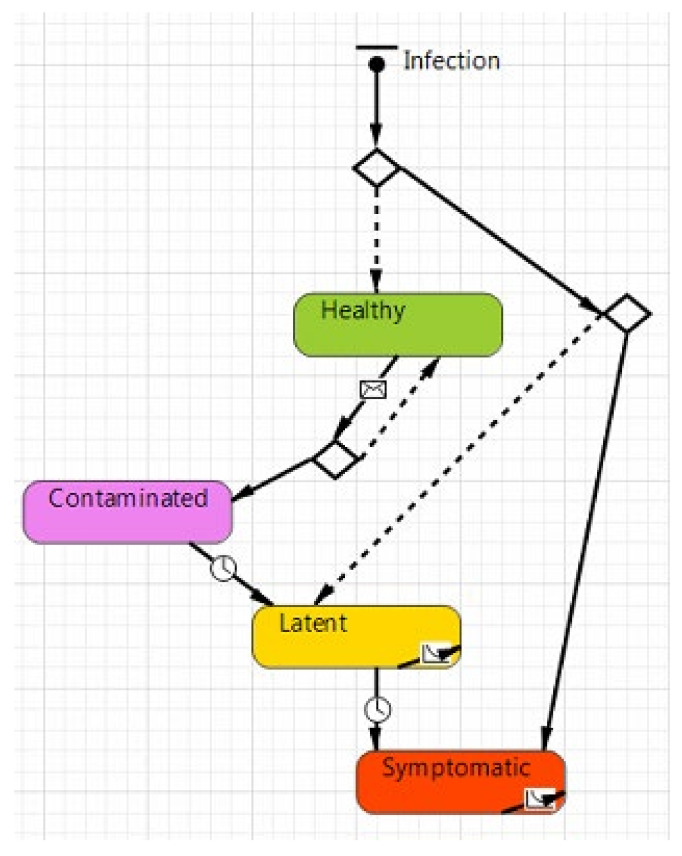
The agent-based model in Anylogic.

Starting from the general SEIR model, in the Agent-Based simulation developed in this study with Anylogic 8.7.1 software, the infection has been modeled with the following states (Figure 3):

- *Healthy* (represented by a green color), in this state there are all those individuals who have not contracted the virus and are in good health;
- *Contaminated* (represented by purple color), which is the state of individuals that are infected during the simulation run but cannot yet contaminate other individuals;
- *Latent* (represented by yellow color), in this state all those individuals have contracted the SARS-CoV-2, but the disease does not manifest itself with any external signs or symptoms. However, latent individuals are contagious;
- *Symptomatic* (represented by red color), in this state there are all those individuals that are contaminated and can contaminate other people. They differ from the previous state because these show evident symptoms, such as fever.

The structure is, therefore, very similar to a traditional SEIR compartmental study. The most significant difference from the SEIR model is that in this case, the cured people are not taken into consideration, because it is assumed that the population under study is ‘new’ each day but also because there are no researches that ascertain the fact that cured people are immune to the disease [52]. Moreover, the spread of SARS-CoV-2 is considered possible only through the direct interaction of individuals, under the minimum interpersonal distance of 1 m, for almost 15 min [53]. This interaction triggers the transition from the state Healthy to the state Contaminated. The probability of contamination (Contagion Probability) has been calculated using the simulator provided by the Max Planck Institute to estimate the aerosol infection risk in an indoor environment [54]. Indirect transmission of SARS-CoV-2 (such as transmission through contaminated surfaces) is not taken into account. The other transition between states is defined through the parameters and variables showed in Table 5.

### 4.2. People Flow

**Figure 4:**
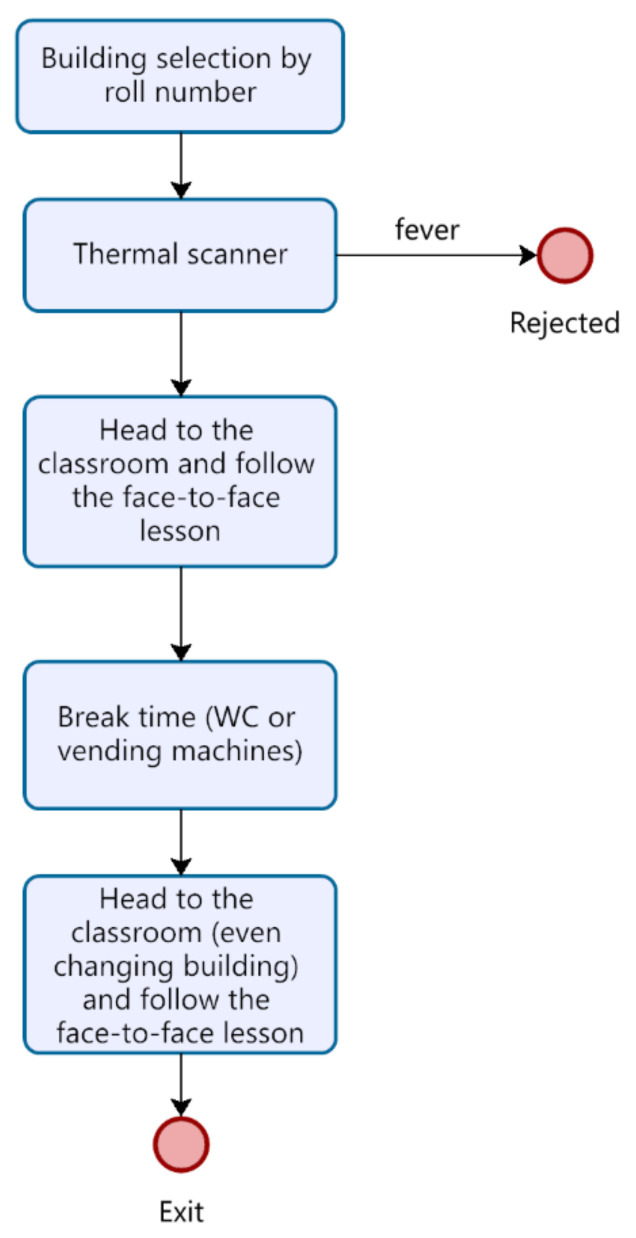
Flow chart regarding the student’s flow.

**Figure 5:**
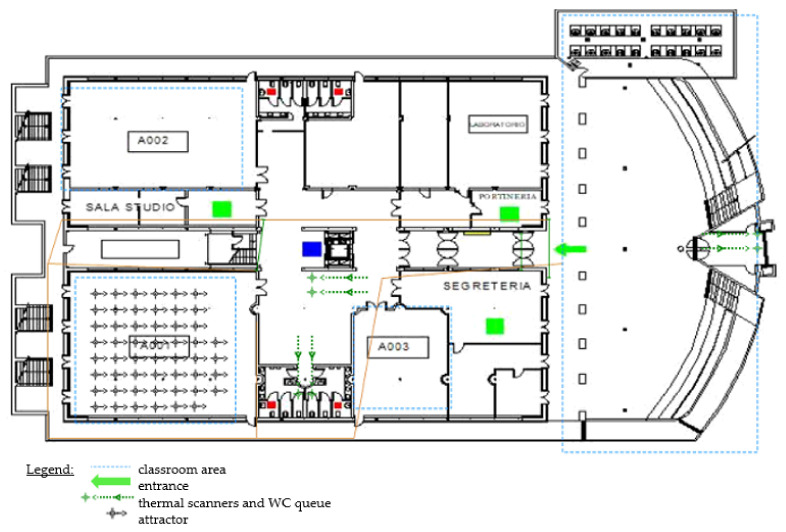
DES model floor.

To depict the flow of people in the model, a DES model has been provided. In DES, the actions undertaken by entities are guided by events that occur during the time. The flowchart, represented in Figure 4, reports the main events that define people’s movements in the settled environment of the DES. The test being case-developed in a University campus, the main activities concern the lectures held in the classrooms and the breaks during the lessons, including the canteen service at lunch break. In Anylogic, the entities (i.e., the students) flowing in the space have been modeled as pedestrians walking through the University campus, included as CAD drawings on the model floor (Figure 5).

**Figure 6:**
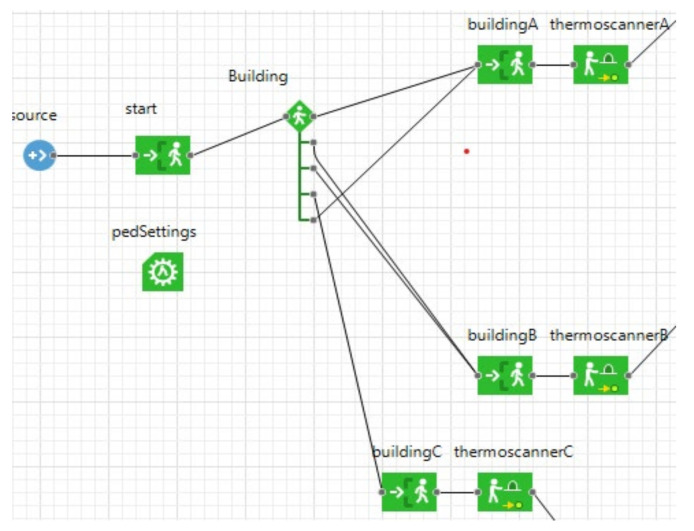
Entities’ creation and entrance.

**Figure 7:**
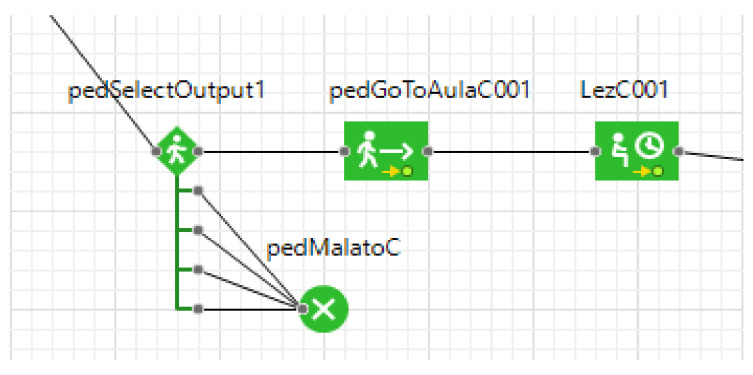
Healthy and infected rules.

The DES starts with creating the entities, which arrive at the University facility where their temperature is checked using the thermal scanners. If the result is a temperature of less than 37.5 °C, they can enter the buildings and are redirected to their classrooms. Otherwise, it is considered a symptom of SARS-CoV-2, and therefore they are rejected (Figure 6 and Figure 7).

**Figure 8:**
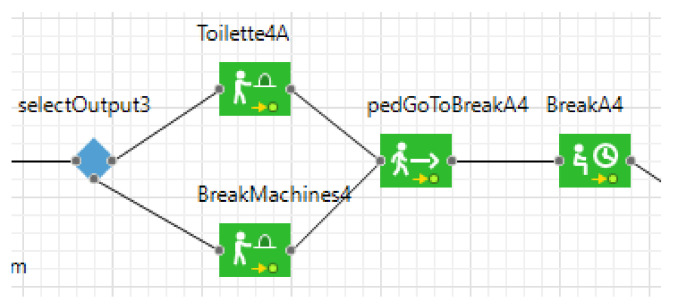
Break time process.

During the break times (Figure 8), students are guided to the vending machines and the bathroom, and always the nearest service is chosen. Then they are redirected to large areas outside the buildings until they have their next lesson. Approximately all students follow the same process during the day, depending on their course schedule. Indeed, each student has a defined lecture schedule based on his/her course and, according to this, the student chooses the path to reach the classroom in which lectures are held.

**Figure 9:**
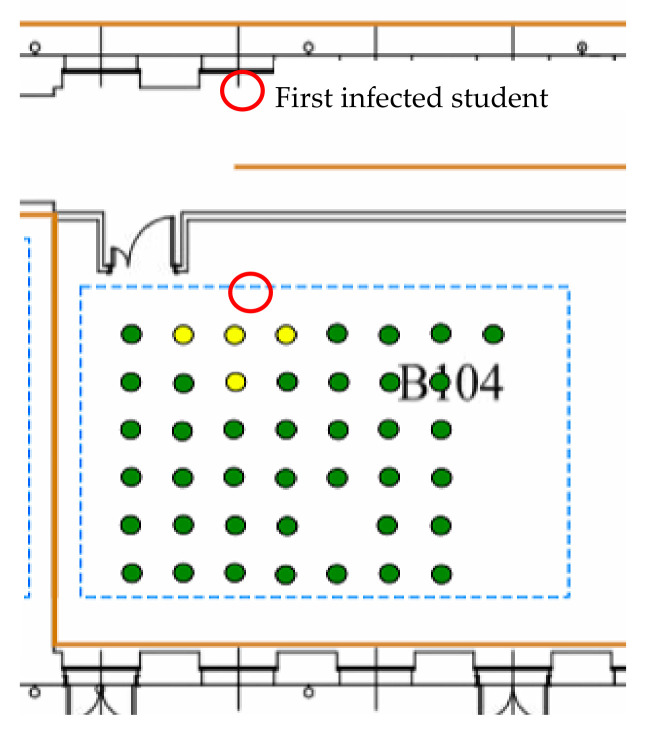
Example of infection in a classroom containing a latent individual.

In the DES people flow model, the position of each student affects the possibility of being infected. The entities of the DES models (i.e., the students) contain the agent-based model that defines their health state and sets the infection mechanism. With this combined approach, the contagion is simulated. In Figure 9, an example of how the contagion occurs is represented. In the red circle, the first latent students entering the classroom are highlighted. The individuals exposed for a defined period of time next to them can be infected. With this mechanism, the contagion can be propagated during the lecture or the break time. 

## 5. The Test Case Analysis: University of Bergamo Case

This section shows the case developed to assess the applicability and validity of the proposed HS model. In the following subsections the case will be described (Section 5.1), and the case validation and results will be discussed (Section 5.2 and Section 5.3).

### 5.1. Case Description

This study took place at the Engineering campus of the University of Bergamo during the 2020/2021 fall semester, that is when the University had adopted containment policies to enable a gradual return to the lecture rooms. The semester consists of 62 days of lectures, but in the AnyLogic model run, it has been decided to consider five days since, as already pointed out, it is assumed that each day the population of students is new, namely new individuals with the related state (healthy, contaminated, latent, symptomatic) enter the system every day according to the probabilities defined. On the University campus, four buildings are present. The maximum capacity of students for each building is reported in Table 6. In order to maintain the correct social distancing, the rules of the University set an occupancy of classrooms equal to 25–30% maximum, trying to minimize the possibility of gatherings as much as possible. Ideally, considering a maximum occupancy of the teaching spaces equal to 25% of their nominal capacity, a limited number of classrooms available for teaching would be obtained. Moreover, other precautionary measures have been settled:

- Body temperature detectors at each entrance;
- The obligation to wear a mask;
- Defined paths to follow to move inside the buildings (entrances and exits);
- Sanitizing gel dispensers in all the most frequented points and the classrooms;
- Indicators for the distancing of the counters with access to the public.

It is also assumed that the social distancing is respected (2 m), but also the correct ventilation of closed places and the proper sanitation of environments and surfaces, according to the WHO indications, are applied. 

Given the classroom availability limitation, only some courses have been considered, and the timetables of the lessons (encompassing breaks) for each course have been included in the Anylogic simulation. Finally, only 192 students, 16 lessons, and 12 classrooms have been considered. Students have been modeled as agents, with the COVID-19 infection model described in the previous section. In each classroom, Anylogic attractors allowed controlling agent location inside the space. Finally, other services available to students such as restrooms, break areas, snack machines, toilette, and even thermal scanners at the entrance to each building have been modeled. Students’ flows have been represented in the simulation, as explained in the previous section.

### 5.2. Model Validation Scenarios

The simulation run has been performed with a Windows 10 Pro PC equipped with a Core i7 3.0 GHz processor with 16 GB of RAM. The simulation duration lasts five days, namely 120 h, to simulate one week of the 2020/2021 fall semester, consisting of 62 days of classes. Five replications have been run for each scenario. The choice of the number of replications has been guided by the suggestions provided by the literature about simulation [60] and has been limited due to the huge computational effort required to run a single replication.

Table 7 summarizes the main results of the Base Scenario described previously, clearly showing that current rules are safe as the number of infected people that have entered in the simulation runs is very small (2.2 infected individuals/week), and this has not given any input for an internal contagion (0.2 contaminated individuals/week). Individuals affected by SARS-CoV-2 are partially ejected at the thermal scanners placed at the entrances while the remaining asymptomatic infected students who enter the buildings fail to infect their friends because 25% classroom occupancy allows for adequate social distancing. Indeed, the number of contaminated people within the University resulting from these simulations is next to zero and, statistically analyzing the simulation outputs, it is possible to say that the contaminated people are a maximum of 0.8 individuals/week within the 95% confidence level.

Even if the model is randomized, the data are rationally consistent with the given inputs: to mathematically calculate the number of infected entrants with the given variables, first of all, the number of daily participants has been multiplied by five days (assumption: 5 days/week), then the result must be multiplied by the contagiousness index, under these circumstances equal to 0.0029. Therefore, 192 students * 5 days * 0.0029 = 2.7 infected individuals. Observing the simulation run, it has been possible to find that contagion does not occur during lectures, but maybe it can occur during breaks or next to entrances/exits.

A sensitivity analysis has been carried out to evaluate the effects produced by changes related to input parameters on the simulation outputs, i.e., to validate the correctness of the model [61]. In particular, sensitivity to inputs can be evaluated simply with the approach “one factor at a time (OAT)” by varying one parameter at a time and keeping the other values fixed and equal to those of the reference [62]. Thanks to this analysis, the decision-making process is improved and simplified, making it possible to assess the robustness of a decision if we consider that a decision is robust when it remains valid as the data on which it made the change. In order to test the robustness of the model under study, another simulation has been executed varying only the input regarding the contagiousness index: it is no longer equal to 0.0029, but now corresponds to 0.0046 (Validation Scenario). It was not chosen at random, but rather the index was calculated without regard to the assumption that only individuals between the ages of 19 and 50 enter the University. Consequently, the calculation made to find the new index was as follows: Total cases in Lombardy/total population in Lombardy in March 2021, which numerically results as 48,559/10,600,000 = 0.004581 = 0.46%. The results of these new simulations (Table 8) lead to similar conclusions as the Base scenario: contaminated individuals within the University during classes remain near zero, so current rules are more than safe, even with this alteration in an input variable. If we consider a 95% confidence level, the maximum number of contaminated individuals is 2.3, so a very limited number of contagions can still be observed.

The change evidenced by the results increases the number of infected individuals at the model entry, but this is justifiable, since the input variable changed (Contagiousness Index) is proportional to the number of those infected. The individuals contaminated inside are little affected by the increased Contagiousness Index.

The goal of this sensitivity analysis, in addition to testing the robustness, is undoubtedly identifying any errors in the model by encountering unexpected relationships between inputs and outputs to gain a greater understanding of the relationships between variables. Mainly, what is evident with this study is a linear correlation between the number of infected individuals and the value of the Contagiousness Index. The index regarding contagiousness defines the proportion between healthy and diseased inputs within the state chart, describing the student in the constructed model.

### 5.3. Case Results—Testing Alternative Scenarios

Different scenarios have been simulated to test the modifications in the measures currently adopted to limit the contagion. Two alternatives have been simulated starting from the scenario in which the contagiousness index is higher (Validation scenario).

First, the social distance was diminished, increasing the occupation of classrooms to 50% in order to force students to stand less than two meters apart. In this scenario (Scenario 1), the number of participants became 384 students per day. To test this change, twice the attractors have been added in each classroom, and the database that describes the entrance of the students in the University has been modified, inserting every potential time of arrival of the 384 participating students. On average, a lesson lasts almost 2 or 3 h; in the constructed model, a 15-min break is set every 45 min of class time. Since contact with other infected people is dangerous after only 15 min, the individuals adjacent to an infected individual can be contaminated at the end of the lesson. Detailed results are reported in Table 9.

Indeed, when doubling the occupation of the classrooms, and therefore allowing more students to attend the lectures, the number of infected individuals within the engineering buildings increases. With an average number of 7.2 infected individuals entering the systems, a similar number of students are contaminated during the week (6). Considering a 95% confidence level, the maximum number of contaminated individuals rises to 11.

In order to test the possibility of increasing the occupancy rate of classrooms to allow more students to take face-to-face lectures, a second scenario (Scenario 2) has been tested, studying the effects of personal protective equipment (PPE). In fact, despite students within the University being required to wear a mask, nothing has been specified about the mask type and the behavior to adopt towards it. Since PPE effectiveness is complete only if the mask is worn correctly (covering both mouth and nose) and hands are washed both before wearing it and before taking it off, every time you have to touch it, these aspects were considered relevant to simulate the contagion.

Masks can be distinguished into three macro groups [63]:

- Surgical masks, which do not adhere perfectly to the contours of the face, so the protection of the wearer is limited, but they prevent the escape of respiratory secretions/droplets to the external environment;
- FFP masks (filtering facepiece particles) protect both the wearer and others, filtering the particles in the air down to a size of 0.6 µm. They filter from 80% (FFP1) to 99% (FFP3) of the particles;
- Non-sanitary masks (i.e., fabric masks) are not considered medical or personal protective equipment.

Surgical masks should be changed every 3/4 h and cannot be reused if disposable. Instead, fabric masks must be washed every day [64]. The particle emission rate depends on expiratory activity, i.e., breathing, talking, coughing, and jaw movement. Indeed, the most invasive activity is coughing or sneezing; nevertheless, larger particles and droplets that result from a human cough or sneeze fall to the ground within a short distance due to gravity, but smaller particles can be carried for greater distances, up to approximately 3 m. Wearing a face mask does not stop 100% of the particles, but it slows down the coughing jets, helping mitigate pandemics associated with respiratory disease such as SARS-CoV-2 [65].

Two insights arise after this:

- The fit of the mask is highly influential on its efficiency (if there is a gap, that is, the mask does not adhere well to the face, then the protection decreases), hence the importance of wearing it in the best possible way;
- Masks can reduce the intelligibility of the speech signal [66] and reduce the intensity of sounds passed through them. As a response, people tend to speak louder, and therefore the number of emitted particles theoretically increases.

According to [67], masks (as well as eye protection and social distance) are factors that decrease the risk of contagion, with a specific ratio according to their characteristics. 

Bringing all this back to the HS model, in Scenario 2, it can be assumed that 100% of the students are now all wearing FFP2 masks, which is the most filtering type. This assumption implies a changed Contagion Probability parameter. From the results (Table 10), FPP2 masks will reduce the number of individuals contaminated inside the university buildings, which are less than one individual/week. Moreover, considering the 95% confidence level, the maximum number of individuals contaminated is 2.4; this result is comparable with the solution of the Base Scenario and Validation Scenario, showing how the requirement to wear protective equipment can be an effective measure to contain the contagion while allowing for higher saturation of spaces.

## 6. Discussion on the Use of Hybrid Modeling to Design People Flow to Minimize COVID-19 Diffusion

The case study demonstrates that the implementation of hybrid simulation modeling, combining agent-based and discrete event simulation, is effective in analyzing the impact of the COVID-19 contagious in closed environments. The scenario analysis allows understanding if the implementation of the containment measures is successful in counteracting the contagious spread, supporting decision-makers in identifying which procedures to adopt.

Starting from the research question “How can simulation support decision-makers in re-organizing people flows and internal processes evaluating the risk of COVID-19 infection?”, it is possible to highlight two main achievements of this study. The first, more related to the case study under investigation, shows how simulation is an effective tool for re-designing people flows and internal processes and assessing COVID-19 containment measures and enables to highlight some lessons learned that can be used as starting point from the decision makers. The second result, more technical, shows how adopting a hybrid simulation approach permits better and more appropriate modeling of the phenomenon.

In the case under investigation, different people flows and internal processes have been re-designed as containment measures, considering different epidemiological contexts, and their ability to control the spread of the virus has been assessed through the adoption of simulation. As reported in Table 11, different lessons learned can be drawn out. First of all, from the logistics point of view, thanks to the DES part of the HS model, the students’ path inside the environment has been re-designed to limit the number of contacts. The simulation model permitted to assess that the paths defined to enter and exit classes and buildings, as well as quota measures and the access to break areas avoid students to reduce the safety distance and keep the environment safe. Always taking into consideration the flow procedure of the University, the scenario analysis has been demonstrated to be useful in supporting decision-makers to schedule classes and the occupancy percentage of rooms under different external epidemiological situations. Indeed, the increase of the room occupancy from the actual 25% to 50% shows a substantial increase of the spread of the virus, but the adoption of stricter personal protective equipment (e.g., FFP2 masks) can make the University safer.

From a technical point of view, hybrid simulation has proved to be an effective tool from different perspectives:

- *Models segregation*: the flow and the epidemiological models are clearly separated, allowing for a better description of the different aspects via using the appropriate modeling notation (i.e., DES for the people flow and agent-based for the person contagious spread).
- *Flexibility*: although in general it would be possible to model the agent (i.e., students) flow and the contagious infection spread using only DES through a long and complex sequence of ‘if-then’ blocks, attributes and variables, the segregation of the models and the adoption of ABS allowed higher flexibility, especially when the agent population and the characteristics of epidemiological model change (e.g., the statechart can be easily modified considering the evolution of the knowledge on COVID-19).
- *Detection of critical situation in advance:* the interactions between agents (i.e., students) in the system (i.e., University) can highlight in advance possible critical situation that may verify in the environment due to the interactions among agents within the population. In summary, the hybrid approach allows for the identification of results and outcomes that would be extremely difficult to detect via a DES or ABS approach alone. The higher the number of students or other variables, the higher the difficulty of the pure DES to identify additional outcomes.
- *Simplicity/effectiveness*: due to its nature, agent-based modeling allows for better and simplified modeling of the compartmental models typically used to model infection spreading, eliminating the necessity of modeling the virus behaviour via complex DES constructs.

## 7. Conclusions

The surfacing of new infectious diseases has become a serious global problem, especially in the last year with the emergence of COVID-19. Enclosed social places such as pubs, shopping centers, schools, and restaurants are recognized in the last period as places of the rapid spread of epidemics. Moreover, in factories and production sites in general, keeping under control the diffusion of SARS-CoV-2 is of utmost importance. Indeed, as it occurs for the application case discussed, i.e., University facilities, everywhere is fundamental to establish the right balance to limit the spread and exploit the structure’s full potential.

The agent-based compartmental model developed in this work describes the main problems of COVID-19 spread in closed places: the contagion is invisible (also due to asymptomatic individuals) and rapid (it is enough a single meeting with an infected individual to be contaminated), and the measures adopted impacts effectively on the virus diffusion. Starting from the settings of these measures, thanks to the HS model constructed through the software AnyLogic, it has been possible to simulate the real behaviors of people in the space. In particular, going beyond the case application and assuming a broader perspective, positioning of facilities and queues, schedule of working activities, entrances, exits, and paths to move inside buildings are only some possibilities in the plethora of decisions that must be taken to face the future of COVID-19 pandemics. Even if an improved situation appears in many countries due to an intensive vaccine program, the COVID-19 pandemic is not finished. In the following months, continuous organization and re-organization of public and private educational and working activities and leisure areas will be required.

The advantages of using the HS model proposed in this study are related mainly to the flexibility in adapting the model to several epidemiological conditions, thanks to the parameters that are set in the agent-based model and to different kinds of facilities, potentially simulating any space in which contacts take place between individuals.

Hybrid simulation enhances the complementarity of the two approaches, i.e., ABM and DES, providing a complete and usable tool to support decision-makers in evaluating different contagion containment measures and simulating correct and incorrect behaviors of individuals who can potentially attend their facilities.

### 7.1. Limitation of the Study

Despite the high potential of applying the proposed HS approach, some limitations characterize this work and should be addressed in the future.

The first limitation of the proposed model is that the population supposed is always new and analyzed only inside the space modeled, i.e., for class hours. Therefore, there is no possibility of considering the individual variation: each participant is equal to the others and is defined from the same variable values. Only the states of entry vary in the model (they can be healthy, latent and symptomatic). For this reason, the model does not allow the evaluation of factors that can be analyzed over time, such as the effect of a quarantine.

Another limitation involves the settings of the contagiousness index and contagion probability, which have been chosen to maintain a cautious attitude, considering the worst-case scenario. Better estimations of these parameters can be achieved by analyzing the epidemiological situation of the specific area in which the simulated facility is located, simultaneously considering the age and job of the individuals, the means of transport they utilize to reach the facility, and so on. Moreover, a more significant statistical analysis of these simulation results could be performed, increasing the number of replications for the simulation runs of each scenario.

Finally, another limitation concerns the contagion mechanism that have been used to replicate the virus diffusion, which is based on the 15 min contact under the safety distance of 2 m. Even if this model is appropriate in many cases (i.e., factories in which spaces are enormous and contagion effectively occur due to the strict contacts between people), other approaches can be used in tiny spaces, such as in classrooms and schools. Since some data are now becoming available about how the droplet diffusion in closed spaces can promote the virus diffusion while social distancing is respected, different ABMs can be provided to take into account this aspect. In addition, the defined parameters are not currently able to capture the presence of several variants at the same time with different contagion logics.

### 7.2. Future Developments

As suggested in Section 7.1, a better integration of data from epidemiological analysis of the population would positively affect the applicability and extension of the model to many other kinds of facilities, providing a well-structured approach to a successful fight against COVID-19 by adopting adequate containment measures.

Furthermore, there are two categories of countermeasures in a pandemic: pharmacological ones and those aimed at limiting contact between people, or limiting contagion through precautions. In this work, we developed an analysis only in the second category, but to have a more complete and current vision, we could include within the study the influence of vaccines and the rate of the administration of them.

What is currently known from the literature is that clinical trials of vaccines have demonstrated a maximum efficacy at most 15 days after the second dose and that it is still debated if the vaccinated individual can no longer be affected by SARS-CoV-2, but probably they can transmit it to other people [68]. Therefore, further developments of the ABM should involve a new state representing these vaccinated individuals. The objective of this possible future study could be to identify the minimum percentage of vaccinated people required to set the occupation of a closed space, avoiding contagion even by not respecting the safety distance, since vaccines are expected to affect the model parameters, decreasing the infection probability.

## Figures and Tables

**Table 1:** Epidemiological studies reviewed.

Paper ID	Title	Reference	Epidemic	Epidemiological Approach
1	Towards a comprehensive simulation model of malaria epidemiology and control	[6]	Malaria	EIR
2	A model simulation study on effects of intervention measures in Wuhan COVID-19 epidemic	[7]	Sars-CoV-2	Ad hoc infection model
3	Mathematical Modeling of Epidemic Diseases; A Case Study of the COVID-19 Coronavirus	[8]	Sars-CoV-2	SIR
4	Epidemic trend and transmission risk of SARS-CoV-2 after government intervention in the mainland of China: A mathematical model study	[9]	Sars-CoV-2	SEIPQR
5	Parameter identification for a stochastic SEIRS epidemic model: case study influenza	[10]	Flu	SEIRS
6	Using simulation technology to analyze the COVID-19 epidemic in Changsha, Hunan Province, China	[11]	Sars-CoV-2	SEIAR
7	An agent-Based Simulation of the Spread of Dengue Fever	[12]	Dengue fever	SEIR
8	Mathematical Modeling of the impact of Malaria Vaccines on the clinical epidemiology and natural history of Plasmodium falciparum malaria: overview	[13]	Malaria	EIR
9	Predictability and epidemic pathways in global outbreaks of infectious diseases: the SARS case study	[14]	SARS	SIR
10	Factors that make an infectious disease outbreak controllable	[15]	SARS	Ad hoc infection model
11	The effectiveness in Contact tracing in Emerging Epidemics	[16]	SARS	Ad hoc infection model (based on [15])
12	Predictive models of control strategies involved in containing indoor airborne infections	[17]	SARS	Ad hoc infection model (partially based on [15])
13	Timely identification of optimal control strategies for emerging infectious diseases	[18]	SARS	SEIR
14	Comparing nonpharmaceutical interventions for containing emerging epidemics	[19]	SARS & MERS	SEIR

Acronyms: SIR = Susceptible-Infectious-Recovered; SEIR = Susceptible-Exposed-Infected-Recovered; EIR = Entomologic Inoculation Rate; SEIPQR = Susceptible S-latent E-infectious I-suspected P-diagnosed Q-recovered R; SEIRS = Susceptible-Exposed-Infectious-Recovered-Susceptible; SEIAR = Susceptible-Exposed-Infected-Asymptomatic-Recovered.

**Table 2:** Comparison of SARS and SARS-CoV-2 Epidemiological studies.

Paper ID	Reference	Asymptomatic Transmission Ratio	Quarantine Delay	Isolation Delay	Quarantine Efficiency	Isolation Efficiency
2	[7]	Not Included	No	No	No	No
3	[8]	Included	Yes	Yes	Yes	Yes
4	[9]	Not Included	Yes	Yes	Yes	Yes
6	[11]	11, 10%	No	Yes	No	Yes
9	[14]	Not Included	No	Yes	No	Yes
10	[15]	11%	Yes	Yes	Yes	Yes
11	[16]	Not Included	No	Yes	No	No
12	[17]	11%	Yes	No	Yes	No
13	[18]	Included	Yes	Yes	Yes	Yes
14	[19]	Included	Yes	Yes	No	Yes

**Table 3:** Related works on simulation applied to COVID-19 pandemic.

Study	Simulation Technique	Objective	Aims			
			Model a Variety of Possible Individual Infection Phases/Interactions	Describe Inner Behavior Uncertainties for the Individual	Model Individual Actions and Organizational Processes in Time and Space	Monitor and Evaluate the Spread of the Contagion within Different Control Policies
[26]	ABM-DES-SD-HS	Suggesting the right simulation technique to support public health decision-making	N.A	N.A	N.A	N.A
[41]	ABM	Illustrating the dynamics of COVID diseases to identify places which have a higher probability to become infection hubs	X	X		
[42]	SD	Simulating the COVID-19 spread in India	X			X
[43]	ABM	Evaluating the virus containment measures in closed environments	X	X		X
[44]	SD	Evaluating the effectiveness of various policies in mitigating transmission and the resulting economic burden	X			X
[45]	HS (ABM-SD)	Evaluting the impact of social distancing measures and presymptomatic and surface transmission on the COVID spread.	X	X		X
[46]	DES	Supporting short-term planning decisions about hospital resources utilization			X	
[47]	DES	Understanding the triage efficacy in the intensive care admission and discharge dynamics			X	X

**Table 4:** General model variables.

Variable	Domain/Unit	Definition
β—Contagion probability	0–100%	The probability an individual can be infected while exposed
α—Initial exposed	0–100%	The percentage of individuals who could be exposed at the starting of the simulation
γ—Initial infectors	0–100%	The percentage of individuals who could be infectors at the starting of the simulation
δ—initial recovered	0–100%	The percentage of individuals who could be recovered at the starting of the simulation

**Table 5:** Agent-based model variables.

Variable	Value	Definition	Source
Contagiousness Index	0.0029	It defines the ratio of infected individuals; it is calculated as the number of positives/Total people in the population, considering the Lombardy Region and people aged 19–50 in February 2021.	[55,56]
Symptomatic ratio	40%	Infected participants are divided into “Symptomatic” and “Latent” based on this parameter.	[57]
Incubation period	Normal Min: 2.33 days Max: 17.6 days Median: 5.41 days Mean: 6.38 days	It is the time from exposure to the causative agent until the first symptoms develop (from “Latent” to “Symptomatic” state).	[58]
Contagion probability	Triangular Min: 0, 11 Max: 0, 26 Mode: 0, 17	It is the probability of being contaminated when in contact with an infected individual (transition from “healthy” to “contaminated” states). It has been calculated considering interpersonal distance equal to 2 m and a different Personal Protective Equipment (e.g., FFP1, FFP2, FFP3 masks).	[59]

**Table 6:** Building’s occupation.

Building	Classrooms	Max. Capacity	Allowed Capacity
Building A	11	1,053	183
Building B	11	493	44
Building C	2	210	45
Building D	3	540	66

**Table 7:** Base scenario results.

Base Scenario: Contagiousness Index = 0.0029		
Run	Individuals Infected from Outside	Individuals Contaminated Inside
1	0	0
2	4	0
3	3	0
4	3	0
5	1	1
Mean	2.2	0.2

**Table 8:** Validation scenario results.

Validation Scenario: Contagiousness Index = 0.0046		
Run	Individuals Infected from Outside	Individuals Contaminated Inside
1	5	3
2	1	0
3	4	0
4	5	0
5	4	0
Mean	3.8	0.3

**Table 9:** Scenario 1 Results.

Scenario 1: Classroom Occupancy = 50%		
Run	Individuals Infected from Outside	Individuals Contaminated Inside
1	7	6
2	5	8
3	11	3
4	7	12
5	6	2
Mean	7.2	6

**Table 10:** Scenario 2 Results.

Scenario 2: FFP2 Masks		
Run	Individuals Infected from Outside	Individuals Contaminated Inside
1	9	0
2	6	0
3	4	0
4	8	1
5	7	3
Mean	6.8	0.8

**Table 11:** Containment measure assessed throughout simulation and lesson learned.

Containment Measures	Example of Measures	Lessons Learned
Flow management	New path, break areas. Closing/opening community services (e.g., canteen, libraries, laboratories)	The paths and the area defined impact the safety distance among students. Introduction of fixed paths, regulation of access to break areas and closure of community services are resulted to be effective measures.
Number and schedule of inputs	Number of classes in presence	The limited number of classes in presence allows better management of flows and common spaces to keep the university safe.
Occupancy rate	Occupancy rate of classrooms	The increase in the occupancy rate must be carefully assessed considering the external epidemiological situation because it creates a multiplier effect in the spread of the infection.
PPE to be adopted	FFP2 vs. chirurgical mask adoption	The massive adoption of FFP2 is effective in counteracting the spread even when the number of students in presence increases.
